# Efficacy and safety of antibiotic therapy for post-Lyme disease? A systematic review and network meta-analysis

**DOI:** 10.1186/s12879-023-07989-4

**Published:** 2023-01-12

**Authors:** Xiaoqian Zhang, Yuwei Jiang, Yihan Chen, Jiaru Yang, Xiaoqi Zhang, Lu Xing, Aihua Liu, Fukai Bao

**Affiliations:** 1 Department of Stomatology, Haiyuan College of Kunming Medical University, Kunming, 650000 China; 2 Medical Microbiology and Immunology Teaching and Research Section, Haiyuan College of Kunming Medical University, Kunming, 650000 China; 3 The Institute for Tropical Medicine, Faculty of Basic Medical Science, Kunming Medical University, Kunming, China; 4 Yunnan Province Key Laboratory of Children’s Major Diseases Research, The Affiliated Children’s Hospital, Kunming Medical University, Kunming, China; 5 Department of Orthodontics, West China Hospital of Stomatology, State Key Laboratory of Oral Diseases, National Clinical Research Center of Oral Diseases, Sichuan University, Chengdu, China

**Keywords:** Antibiotics, Post-treatment Lyme disease, Meta-analysis, Treatment, Curative effect

## Abstract

**Background:**

At present, the pathogenesis of post-treatment Lyme disease (PTLDS) is not clear, so the treatment scheme of PTLDS, especially antibiotic treatment, is still controversial. This study aims to evaluate the efficacy of antibiotics in the treatment of PTLDS using network meta-analysis (NMA).

**Methods:**

Following PRISMA guidelines, a systematic literature search was conducted on randomized controlled trials in PubMed, EMBASE, Web of Science and Cochrane Library (the literature was published from database inception through December 16, 2022). Using random effect model and fixed effect model. STATA17.0 software was used to evaluate the quality and heterogeneity of the included research literature.

**Results:**

The system included 4 randomized controlled trials (485 subjects). The network meta-analysis showed that ceftriaxone had better results than placebo [Mean = 0.87, 95% CI (0.02, 1.71)] and doxycycline [Mean = 1.01, 95% CI (0.03, 1.98)] in FSS scale scores. There was no statistical difference in FSS scale scores of other drugs after treatment. In terms of FSS score results, Ceftriaxone was the best intervention according to the SUCRA value of each treatment (97.7). The analysis of outcome indicators such as Beck Depression Inventory (BDI), Mental-health Scale and Physical-functioning scale showed that there was no statistically significant difference between the antibiotic group and placebo group.

**Conclusion:**

Ceftriaxone treatment may be the best choice for antibiotic treatment of PTLD, which provides useful guidance for antibiotic treatment of PTLD in the future.

**Supplementary Information:**

The online version contains supplementary material available at 10.1186/s12879-023-07989-4.

## Introduction

Lyme disease (LD) is an infectious disease caused by Borrelia burgdorferi. Its clinical manifestation is mainly nervous system damage. At the moment, the clinical evaluation and treatment of persistent symptoms after treatment of Lyme disease have not been unified [24, 30]. Guedj et al. Proposed that functional neuroimaging can be used for auxiliary diagnosis and treatment of PTLDS patients with somatic disorders [10]. Ranque-Garnier et al. Proposed that genetics could help the treatment of PTLD [23]. Klempner et al. Proposed that neurotrophic drugs can also be used to reduce the nerve injury caused by nerve symptoms from PTLDS [17]. However, the above treatment methods often only alleviate a certain symptom of patients. For those with systemic multiple symptoms, these methods have limitations. At present, antibiotics are mainly used in clinical treatment. However, after receiving regular antibiotic treatment, a few LD patients will have progressive adverse physiological reactions such as fatigue and pain for more than 6 months, as well as cognitive and memory disorders Lyme disease (LD) is an infectious disease caused by Borrelia burgdorferi. Its clinical manifestation is mainly nervous system damage. At present, antibiotics are mainly used in clinical treatment. However, after receiving regular antibiotic treatment, a few LD patients will have progressive adverse physiological reactions such as fatigue and pain for more than 6 months, as well as cognitive and memory disorders[2]. The Infectious Diseases society of America calls Post-treatment Lyme Disease (PTLDS). PTLDS can damage the physical function, social and psychological of patients [29]. Patients often have problems with memory and attention [31], or their sleep is affected by pain, fatigue, anxiety and other symptoms [28]. Appeal symptoms have a negative impact on their memory, emotional processing and learning [9, 28], which has seriously affected the daily life of patients.

Due to the lack of clinical trials using the above scheme, its safety and effectiveness need to be considered. At present, antibiotic therapy is still widely used in clinic. In addition to the commonly used antibiotics such as penicillin, cefotaxime, doxycycline and clarithromycin [11], some scholars have proposed that dapsone is also effective in the treatment of PTLDS in recent years [2, 15]. Through the review, it is found that the experimental research on the treatment of PTLDS with antibiotics often only obtains the effectiveness of a certain kind of antibiotic treatment by comparing a certain kind of antibiotics with placebo or by comparing two kinds of antibiotics, which makes the scheme of antibiotic treatment of PTLDS have a variety of selectivity. At the same time, there are also cases where the treatment opinions are inconsistent due to the insufficient sample size of the experiment. However, antibiotic treatment also has some disputes. The etiology of PTLDS is still unclear [15, 30]. Therefore, PTLDS is defined as a symptom that persists after regular antibiotic treatment which seems to contradict the conclusion that antibiotic treatment of PTLDS is effective [1]. At present, there is also controversy about whether to extend antibiotic treatment [1, 3, 11, 30, 32]. However, there seems to be no better treatment than antibiotics, and some patients urgently need to solve the pain caused by the disease. Therefore, this paper will conduct meta-analysis on the RCT of antibiotic treatment of PTLDS in the past, analyze the effectiveness of antibiotic treatment of PTLDS. And compare the effectiveness and safety of different antibiotics in the treatment of PTLDS with Network Meta-analysis, hoping to find out the better scheme suitable for the treatment of PTLDS and provide useful guidance for the antibiotic treatment of PTLDS in the future.

## Methods

This study followed the Preferred Reporting Items for Systematic Reviews and Meta‐Analyses (PRISMA) statement checklist [22] and the Cochrane Handbook of Systematic Reviews [13]. This system evaluation program is registered on PROSPERO under the registration number CRD42021238996.

## Search strategy

This study was searched in 6 major databases including PubMed, Embase, Web of Science, Cochrane Library, China National Knowledge Index, and Wan fang by using subject terms and free terms. The search terms included: antibiotics, efficacy, drug therapy, randomized controlled trials, post-Lyme disease syndrome, post-Lyme disease. The literature included in the search was published from database inception through December 16, 2022. Language restrictions are Chinese and English. The search strategies and specific search date are detailed in Additional file 1: Table S3.

## Inclusion and exclusion criteria

Inclusion criteria: (1) randomized controlled trials; (2) patients with post-Lyme disease syndrome which has persisted for at least 6 months after treatment of the initial infection and IgG Western blot positive; (3) patients aged 18 years and above; (4) number of cases providing valid data to measure outcomes; (5) studies that the control group used placebo, while the observation group took the antibiotic for the treatment of post-Lyme disease syndrome.

Exclusion criteria: (1) duplicate studies; (2) purely descriptive studies without control groups; (3) types of studies were empirical summaries, theoretical summary discussions, case reports, animal experiments, and reviews; (4) data were incomplete and not available from the original authors; (5) original texts were not in English or Chinese.

## Study selection

This study was conducted with reference to the PRISMA flow chart [22]. Three researchers (X.Z, Y.J and Y.C) independently conducted the screening of the research literature. Articles were first screened based on the title and abstract, and then the literature was re-screened by reading the full text. In case of disagreement among the three researchers, a third researcher (F.B) was consulted for their opinion.

## Data collection

Three researchers (X.Z, Y.J, and Y.C) used an independent method to extract data predetermined extraction tables and resolved disagreements by: consensus or consultation with third-party researchers (F.B). Extracted data included: (1) basic information; (2) baseline characteristics of participants and inclusion/exclusion criteria; (3) details of the intervention and control group; and (4) outcomes (providing continuous data means, standard deviations (SD), and total number of participants per group). The primary outcome indicators of this study were clinical outcomes after antibiotic treatment, specifically patient scores on the Fatigue measure (FSS-11), Beck Depression Inventory (BDI), Physical-functioning scale, Mental-health scale Results. Valid cases were defined as patients with a significant change in these scales compared to the previous scored results. Secondary outcomes were: incidence of adverse effects (AE).

## Risk of bias

Three investigators (X.Z, Y.J and Y.C) independently performed the risk bias assessment for inclusion in the study. Disagreements were resolved by negotiation or by consulting third-party researchers (F.B). Risk of bias was assessed in the literature of included studies using the Cochrane risk of bias (RoB) assessment method for random sequence generation, allocation concealment, participant and personnel blinding, personnel blinding outcome assessment, incomplete outcome data, selective outcome reporting, and other biases.

## Data synthesis and analyses

We performed the meta-analysis using STATA version 17.0 software. The effects of the interventions were significantly ranked by the surface under the cumulative ranking curve (SUCRA) curves based on the values of the surfaces below. Selected metrics were count data, while or were used as combined effects, confidence intervals (CI) were set at 95%, and P values < 0.05 were defined as statistically significant. Also, for data that could not be subjected to network meta-analysis, we calculated the standardized mean difference (SMD) using a weighted mean 95% confidence interval for statistical analysis. Statistically significant differences were considered statistically important for bilateral P values < 0.05, and heterogeneity was indicated by I^2^ > 50% indicating the presence of significant heterogeneity. When I^2^ ≤ 50%, the heterogeneity of the two included studies was small, so we used a fixed-effects model for the meta-analysis. When I^2^ > 50%, there was heterogeneity in the two included studies, so we performed a meta-analysis using a random-effects model.

## Results

### Results of study selection

**Fig. 1:**
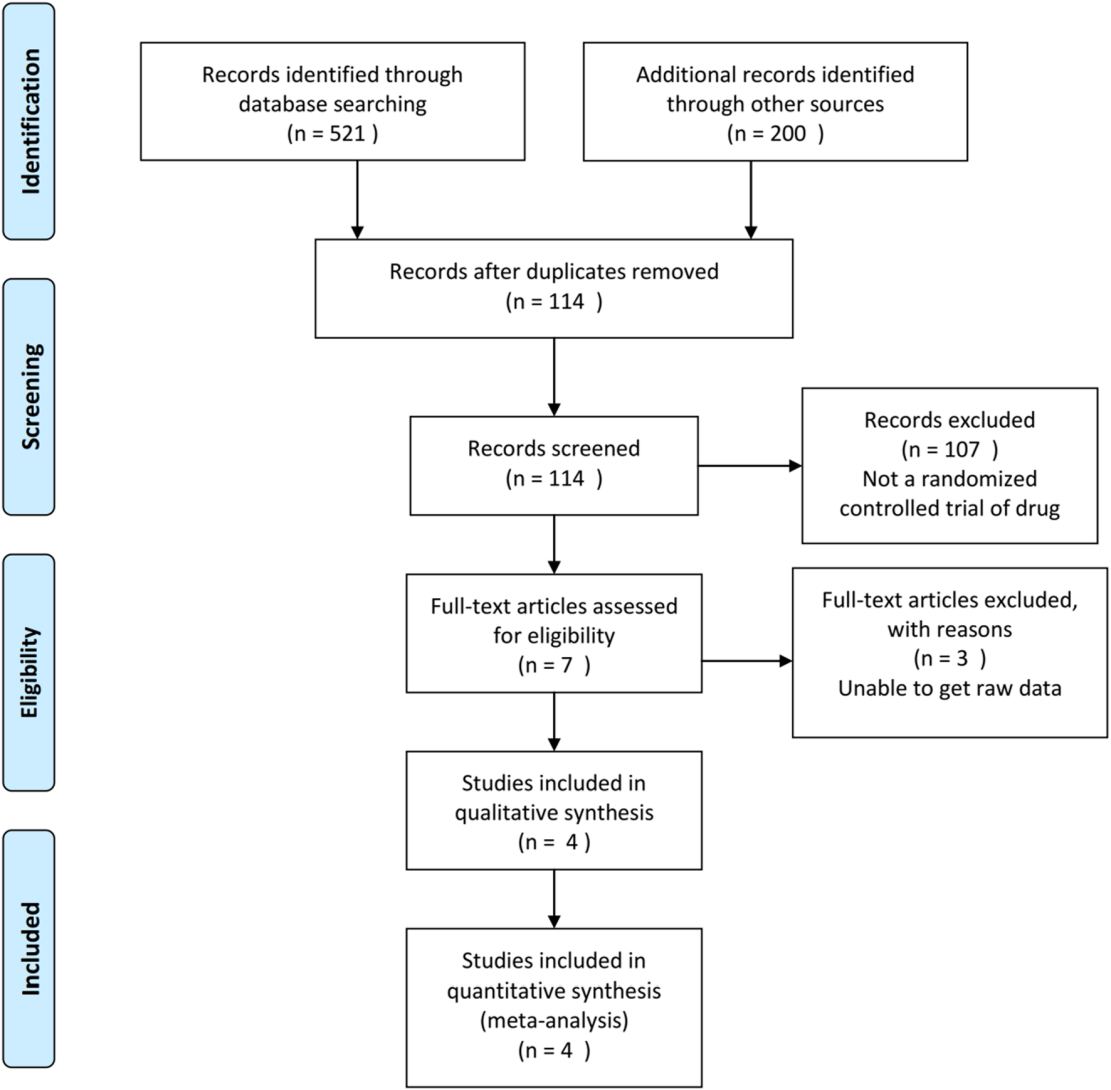
Flow diagram of the selection details of included publications

Randomized controlled trials from database inception through December 16, 2022 were searched in PubMed, Embase, Web of Science, and the Cochrane Library, resulting in a total of 721 articles (including 114 duplicates). Other sources are mainly obtained through the reference in our reading literatures. After removing those with incompatible topics, abstracts, and keywords, we excluded 3 articles with incomplete data or failing to obtain them from the original article authors). Finally, a total of 4 articles were included for subsequent Meta-analysis by full-text reading [5, 8, 16, 19]. The specific process is shown in Fig. 1.

### Characteristics of included studies

Four studies were included with a total of 485 patients, all aged > 18 years, were from the same country, all studies were randomized controlled trials, and the intervention in all studies was antibiotic therapy [5, 8, 16, 19]. The basic characteristics are detailed in Additional file 1: Table S1.

### Risk of bias

Of the four studies included [5, 8, 16, 19], four studies were considered low risk for random sequence generation, incomplete outcome data and selective reporting, and only one study for allocation concealment and blinding of outcome assessment were considered low risk (see Table 1 for details).

**Table 1:** Risk of bias of included RCTs

Study	Random sequence generation	Allocation concealment	Blinding of participants	Blinding of personnel	Blinding of outcome assessment	Incomplete outcome data	Selective reporting	Other sources of bias
Fallon 2008	Low risk	Unclear	Unclear	Unclear	Unclear	Low risk	Low risk	Unclear
Anneleen Berende 2016	Low risk	Unclear	Unclear	Unclear	Unclear	Low risk	Low risk	Unclear
Kaplan 2003	Low risk	Unclear	Low risk	Unclear	Unclear	Low risk	Low risk	Unclear
aKrupp 2003	Low risk	Low risk	Unclear	Unclear	Low risk	Low risk	Low risk	Unclear

### Primary outcome

We conducted a network meta-analysis of the Fatigue measure (FSS-11) score results and a meta-analysis of the Beck Depression Inventory (BDI), Physical-functioning scale, and Mental-health scale score results.

#### Network meta-analysis

Fatigue measure (FSS-11).

##### Network diagram of the four drugs (including placebo)

**Fig. 2:**
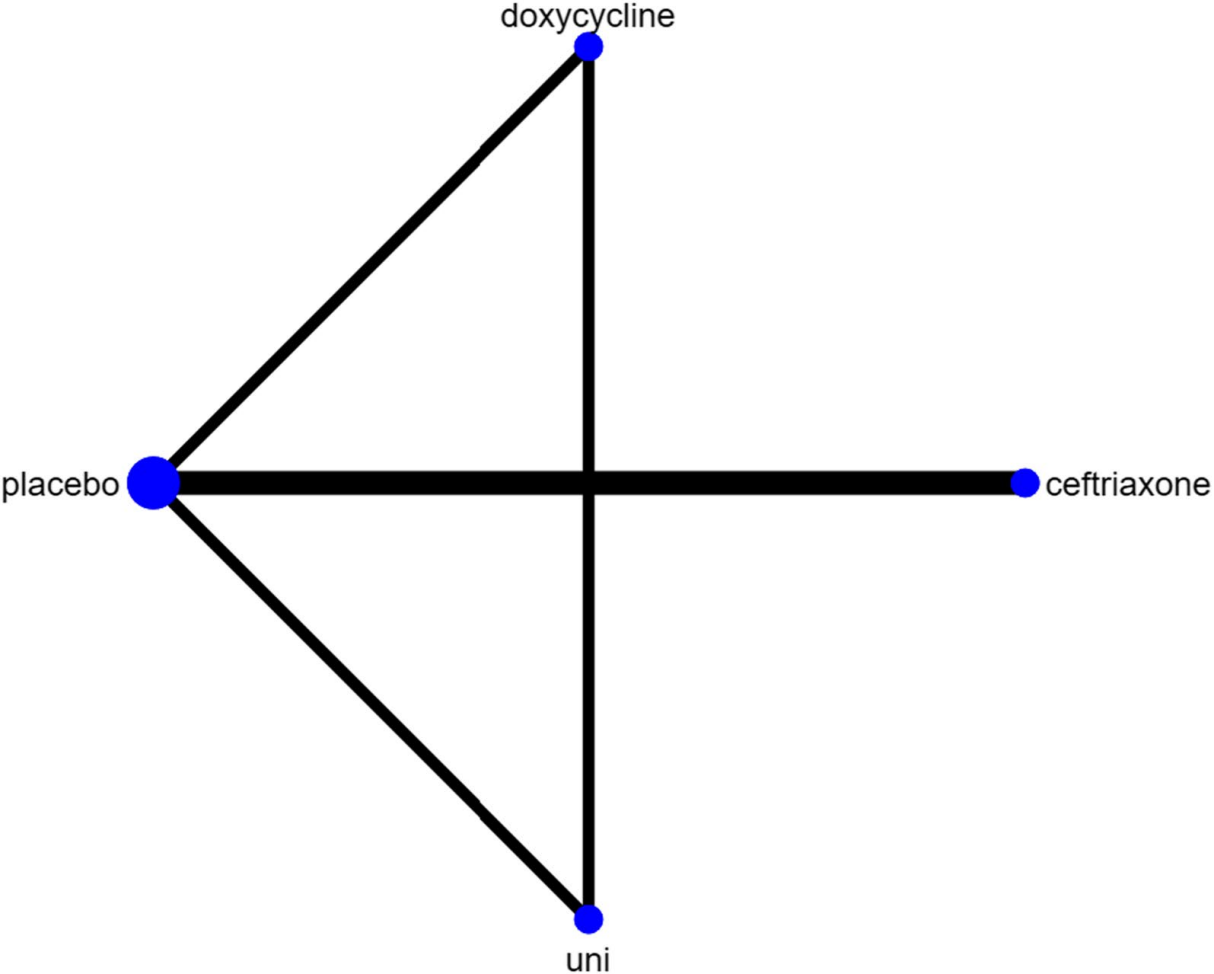
Network plot of different interventions for treatment of post-Lyme disease. The size of the point in the network graph is proportional to the number of subjects, while the thickness of the line is proportional to the number of studies. uni, clarithromycin + hydroxychloroquine

In order to study the effect of different antibiotics on FSS outcome indicators after treatment of PTLD, therefore, this study constructed a network by STATA 17.0. The results of the network analysis showed that the sample size of the trials with direct comparison of placebo and ceftriaxone was the largest, as well as the sample size with placebo (Fig. 2).

##### Confidence interval (CI) Plot

**Fig. 3:**
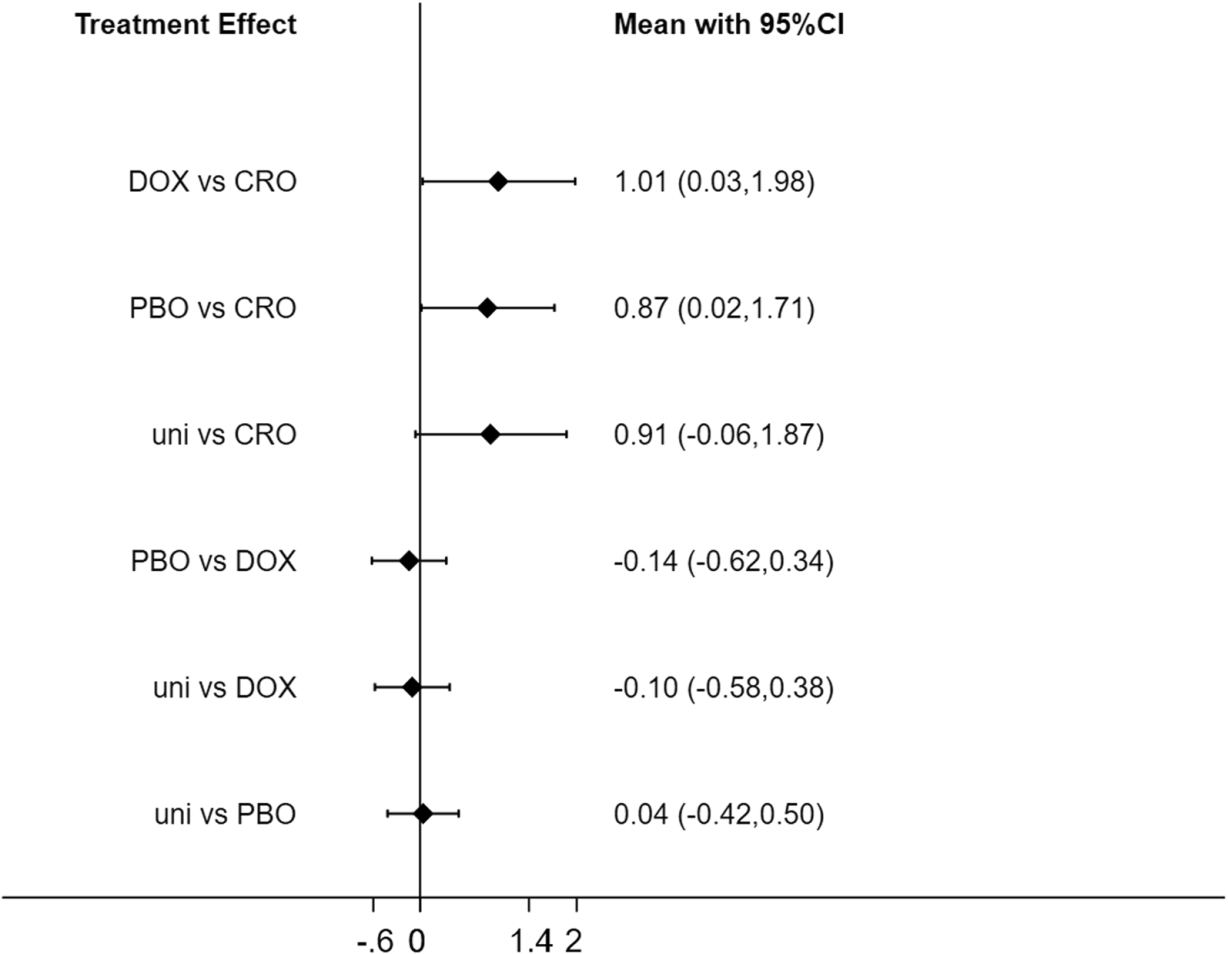
Network plot of different interventions for treatment of post-Lyme disease. Abbreviations: Abbreviations: DOX, doxycycline, CRO, ceftriaxone, PBO, placebo, uni, clarithromycin + hydroxychloroquine

Compared with ceftriaxone, there was a significant difference in the treatment groups of doxycycline [Mean = 1.01, 95%CI (0.03,1.98)], Compared with ceftriaxone, there was a significant difference in the treatment groups of placebo [Mean = 0.87, 95%CI (0.02,1.71)], compared with ceftriaxone, there was not significant difference in the treatment groups of clarithromycin + hydroxychloroquine [Mean = 0.91, 95%CI (− 0.06, 1.87)], compared with doxycycline, there was not significant difference in the treatment groups of placebo [Mean = -0.14, 95%CI (− 0.62, 0.34)], compared with doxycycline, there was not significant difference in the treatment groups of clarithromycin + hydroxychloroquine [Mean = − 0.10, 95%CI (− 0.58, 0.38)], compared with placebo, there was not significant difference in the treatment groups of clarithromycin + hydroxychloroquine [Mean = 0.04, 95%CI (− 0.42, 0.50)]. Because lower FSS scores indicate that the patient's symptoms have improved, where the doxycycline and ceftriaxone comparison showed statistically different FSS scale score results after doxycycline and ceftriaxone treatment, with ceftriaxone having a better FSS Scale score results were better, and the comparison of placebo and ceftriaxone showed statistically different FSS scale score results after placebo and ceftriaxone treatment, and ceftriaxone treatment was better than placebo treatment in terms of FSS scale score results. There was no statistical difference in the FSS scale score results after treatment for the remaining drugs when compared two by two (Fig. 3).

##### Publication bias

**Fig. 4:**
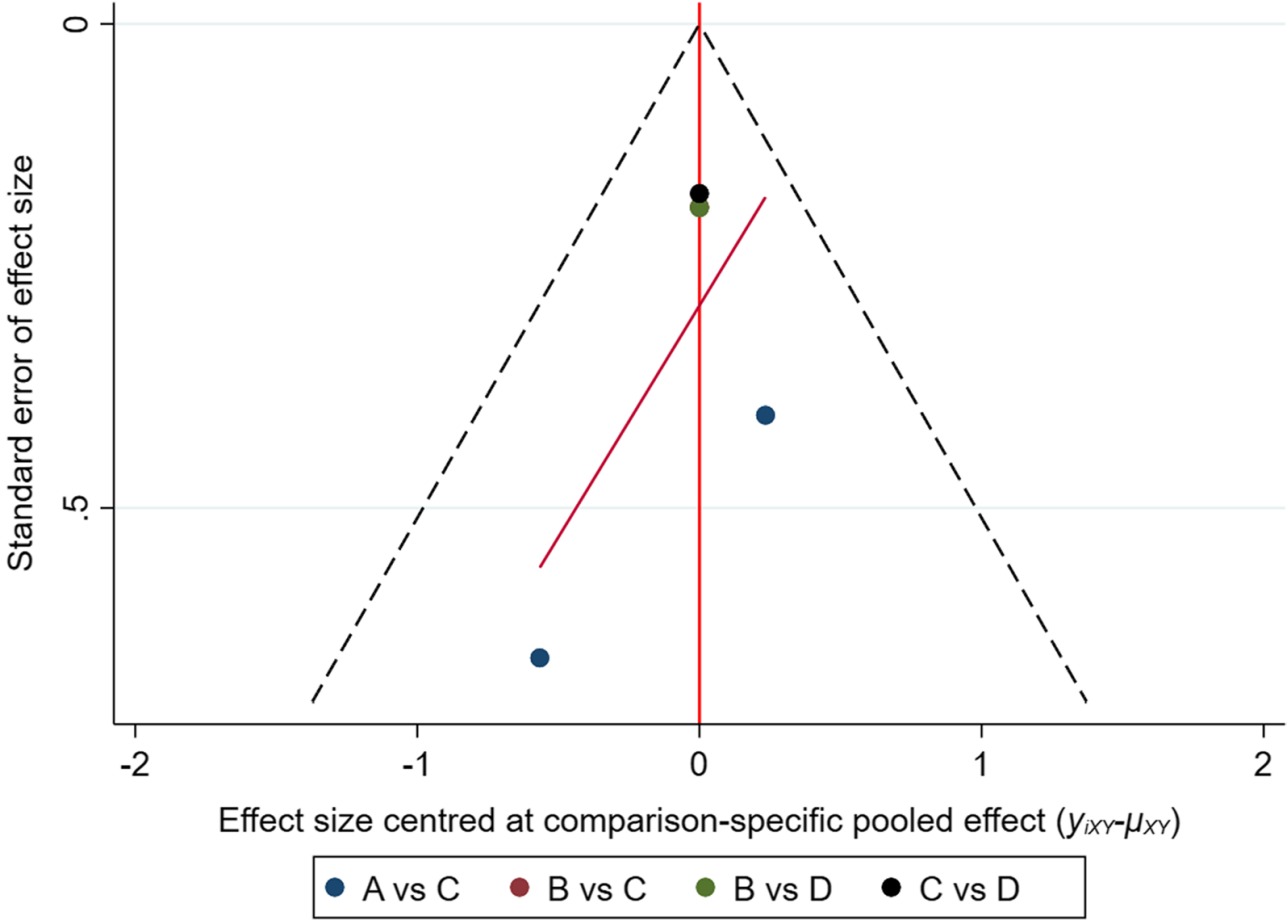
Funnel plot for publication bias in selected studies. Abbreviations: A = ceftriaxone, B = doxycycline, C = placebo, D = clarithromycin + hydroxychloroquine

We analyzed publication bias by funnel chart. Regarding publication bias, all outcome studies in the report were almost symmetrically distributed, suggesting that there may be no publication bias (Fig. 4).

##### Ranking plot

**Fig. 5:**
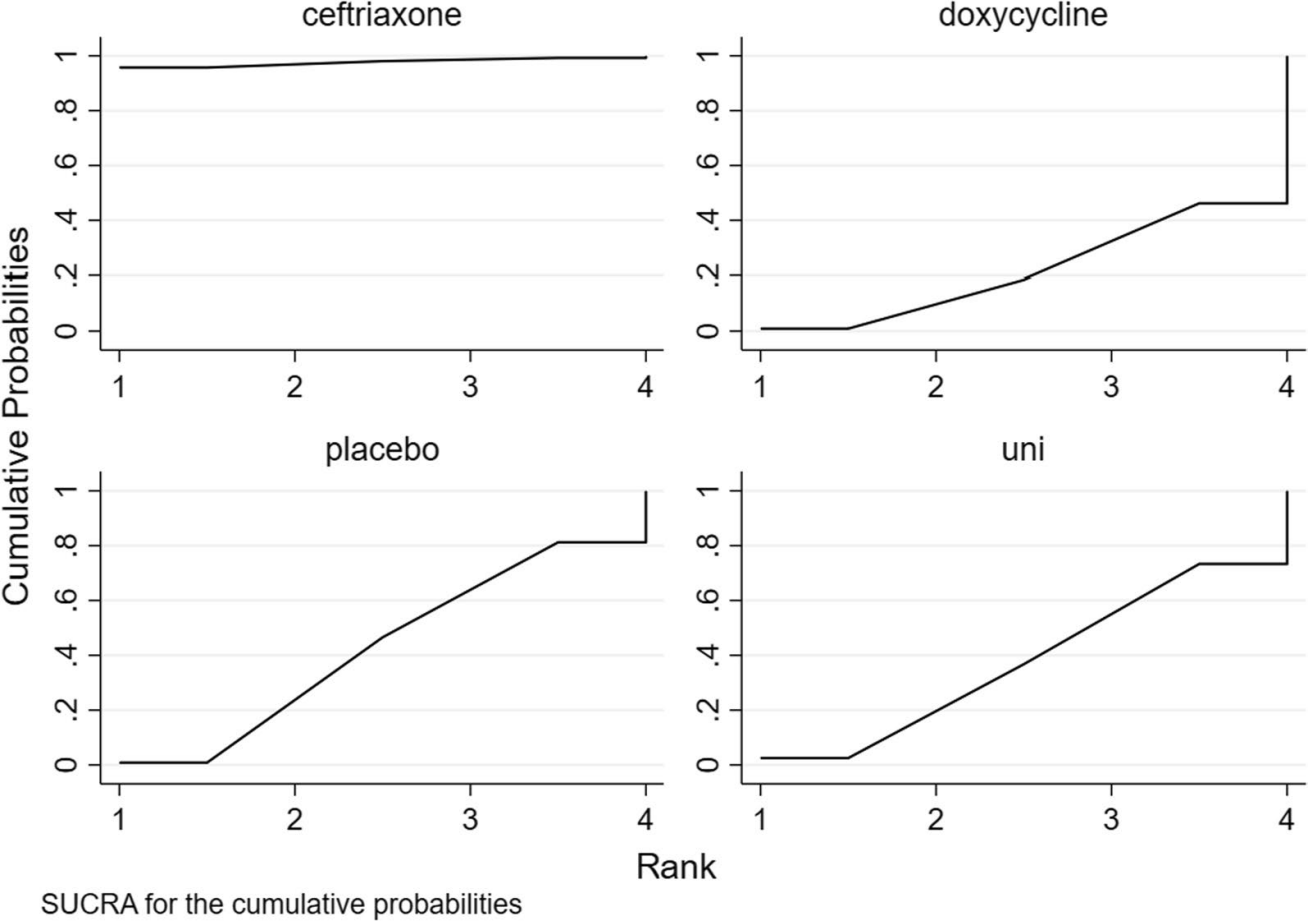
SUCRA for the cumulative probabilities. SUCRA rankings of antibiotic treatment of post Lyme disease syndrome. Abbreviation: uni, clarithromycin + hydroxychloroquine

The percentage efficiency of each treatment is shown in descending order according to the SUCRA value of each treatment ceftriaxone (97.7), placebo (21.9), doxycycline (42.9), clarithromycin + hydroxychloroquine (37.5). Ceftriaxone emerged as the best intervention as far as the FSS scores were concerned (Fig. 5).

#### Meta-analysis

One RCT with three subgroups [5] was regrouped according to the Cochrane Handbook of Systematic Reviews [13], and when combining effect sizes, scholars referred to the different types of antibiotics involved collectively as antibiotics in both retrospective and experimental articles, so we also referred to the different types of antibiotics in the four RCTs [3, 4, 11, 17, 21], and we analyzed data collectively as antibiotics when performing effect size categorization. Our data results used SMD, because the units and time points of measurement indicators are different.

##### Beck Depression Inventory (BDI)

**Fig. 6:**
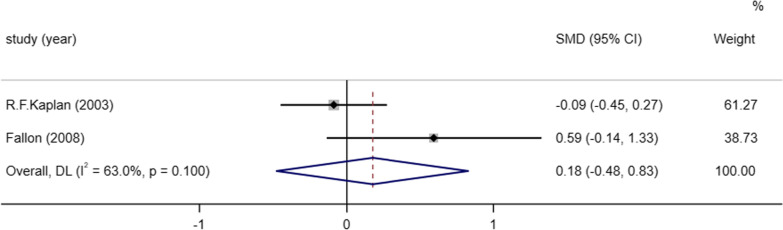
Meta-analysis on the BDI of post Lyme disease syndrome treatment in antibiotic therapy group vs. control group. NOTE: Weights are from random-effects model

The Beck Depression Inventory (BDI) is an outcome indicator used to assess the degree of depression, and two studies [8, 16] reported the results of BDI scores after medication. There was significantly heterogeneity between the two included studies (I^2^ = 63.0%, *P* = 0.100), so we performed a meta-analysis using a random-effects model and the results of the meta-analysis showed that the difference between the BDI score results after treatment and the placebo treatment group was not different statistically significant (SMD = 0.18, 95% CI (− 0.48, 0.83), *P* = 0.598) (Fig. 6).

##### Mental-health scale

**Fig. 7:**
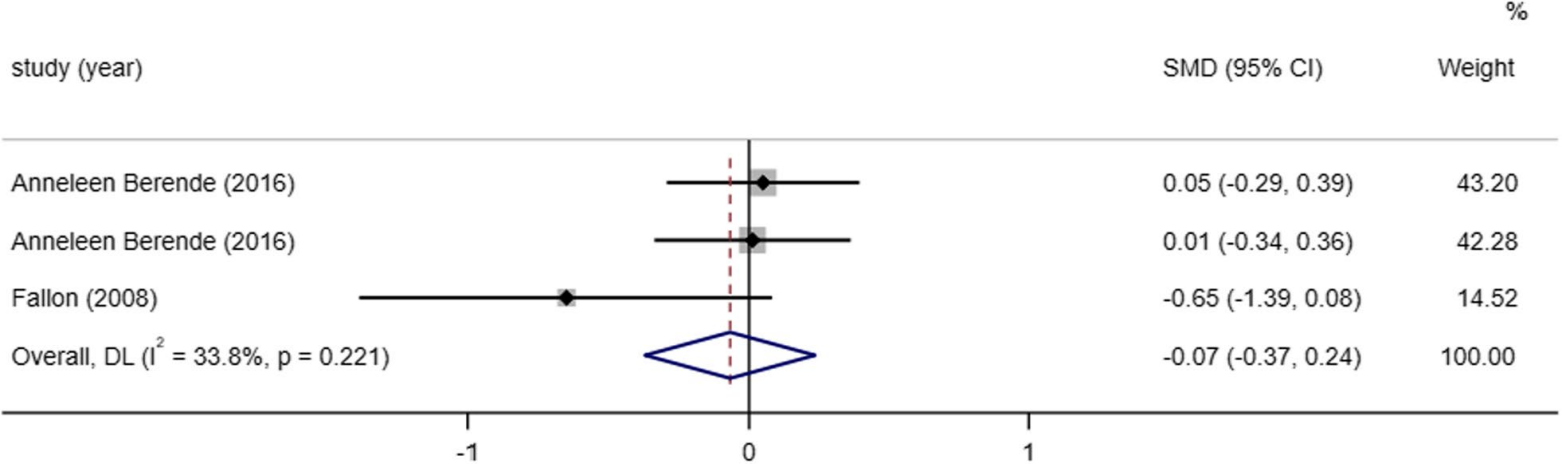
Meta-analysis on the MCS of post Lyme disease syndrome treatment in antibiotic therapy group vs. control group. NOTE: Weights are from fixed-effects model

MCS is an outcome indicator used to assess mental health and two studies [5, 8] reported the results of MCS scores after medication between studies, the heterogeneity of the two included studies was small (I^2^ = 33.8%, *P* = 0.221), therefore we performed a meta-analysis using a fixed effects model and the results of the meta-analysis showed that the difference between the MCS score results after treatment and the placebo treatment group was not different statistically significant (SMD = − 0.07, 95% CI (− 0.37, 0.24), *P* = 0.661) (Fig. 7).

##### Physical-functioning scale

**Fig. 8:**
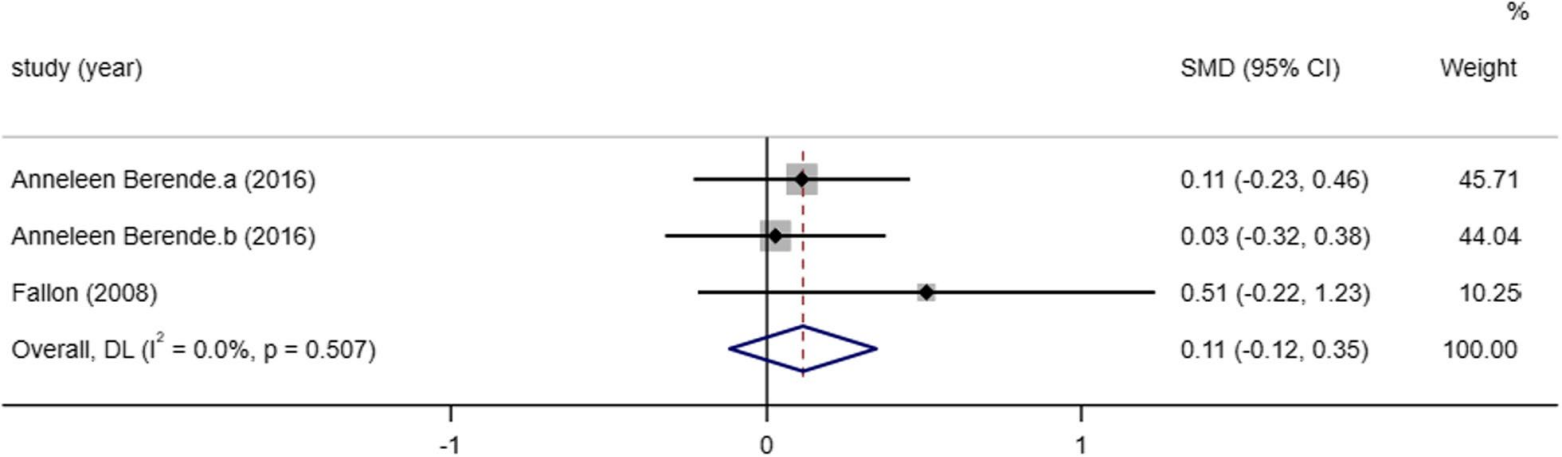
Meta-analysis on the PCS of post Lyme disease syndrome treatment in antibiotic therapy group vs. control group. NOTE: Weights are from fixed-effects model

PCS is an outcome indicator used to assess physiological function and two studies [5, 8] reported the results of PCS scores after medication between studies, the heterogeneity between the two included studies was small (I^2^ = 0.0%, *P* = 0.507), therefore we performed a meta-analysis using a fixed effects model and the results of the meta-analysis showed that the difference between the PCS score results after treatment and the placebo treatment group was not different statistically significant [SMD = 0.11, 95% CI ((− 0.12, 0.35)), *P* = 0.334] (Fig. 8).

### Secondary outcome-rate of adverse effects

As reported in a total of two studies [5, 19] on the occurrence of adverse reactions above, the main adverse reactions were diarrhea, nausea, rash, mucosal fungal infection, photosensitivity, visual impairment, headache, anaphylaxis, IV sepsis. One of the studies [5] reported the highest incidence of adverse events with doxycycline treatment (AE = 45.35%) (Additional file 1: Table S2). Among them, the highest incidence of diarrhea (AE = 9.83%) was reported for clarithromycin-hydroxychloroquine treatment group (Additional file 1: Table S2), nausea (AE = 10.47%) for doxycycline treatment (Additional file 1: Table S2), clarithromycin-hydroxychloroquine group had the highest incidence of rash (AE = 8.33%) (Additional file 1: Table S2), and doxycycline had the highest incidence of mucosal fungal infection (AE = 5.81%) (Additional file 1: Table S2). Doxycycline treated photosensitivity had the highest incidence (AE = 18.6%) (Additional file 1: Table S2), clarithromycin-hydroxychloroquine group had the highest incidence of visual impairment (AE = 4.17%) (Additional file 1: Table S2), clarithromycin-hydroxychloroquine treatment group had the highest incidence of headache (AE = 2.08%) (Additional file 1: Table S2), and one study [19] reported the highest incidence of ceftriaxone treatment with 3.57% incidence of anaphylaxis and placebo with 11.11% incidence of IV sepsis (Additional file 1: Table S2).

## Discussion

The pathogenesis of PTLD is unclear, so the treatment is still controversial. Currently, antibiotic therapy is the most widely used treatment. Often used in the treatment of PTLD clinical antibiotics are doxycycline, doxycycline, penicillin, ceftriaxone, cefotaxime, cefuroxime, dapsone [2, 14, 15]. Although many studies have shown that extending the antibiotic treatment is invalid, there are no better treatments, while patients are really necessary to solve the pain of disease, so we hope to summarize some rules of treatment and medication to provide some basis for subsequent research, through the analysis of antibiotic treatment of RCT to study the effectiveness and safety of antibiotic therapy.

In the current meta-analysis, we summarized the efficacy of antibiotic therapy for PTLD among a large number of participants, and a total of 721 studies were included in our literature screening. Finally, 4 RCTs [5, 8, 16, 19] which indicated by risk assessment were included in the study. Berende et al. evaluated the efficacy of antibiotics by administering oral doxycycline and oral clarithromycin and hydroxychloroquine to PTLD patients in the experimental group, and assessed the patient status from a physiological and psychological multidimensional SF-36. The results showed that treatment had no additional beneficial effect on health-related quality of life [5]. Fallon et al. treated PTLD patients in the experimental group with intravenous ceftriaxone therapy, and evaluated the changes of patients' neurological symptoms through six different areas of Neurocognitive performance tests, so as to evaluate the efficacy of antibiotics. The results showed that intravenous ceftriaxone therapy can improve patients' cognitive function in the short term [8]. Kaplan treated PTLD patients in the experimental group with oral doxycycline, and evaluated the changes of depression and other clinical symptoms by using the baker Depression Scale (BDI) and other tests, so as to evaluate the efficacy of antibiotics. The results showed that antibiotic treatment had no beneficial effect on improving symptoms [16]. Krupp et al. evaluated the efficacy of antibiotics in the experimental group of PTLD patients by intravenous ceftriaxone and by FSS-11 to evaluate the changes in fatigue. The results showed that intravenous ceftriaxone improved the fatigue symptoms of patients [19].

In the 4 RCTs [5, 8, 16, 19] we analyzed, researchers analyzed the symptoms of patients from multiple physiological and psychological dimensions, scored patients' MCS, PCS, BDI and FSS, and evaluated the differences before and after antibiotic treatment. We conducted a further meta-analysis on these scores.

MCS can comprehensively assess individual mental health status, and the survey results can help to understand the mental health status of PTLD patients, and provide the scientific basis for the hospital to formulate relevant mental health promotion programs [5, 6, 18, 20, 30]. A meta-analysis of MCS scores in the two studies [5, 8] using a fixed-effect model showed no statistically significant difference in MCS scores after treatment compared with placebo [SMD = − 0.07, 95%CI (− 0.37, 0.24), *P* = 0.661 > 0.05]. PCS evaluates the physical health status of PTLD patients from the perspective of physical health, and provides the scientific basis for hospitals to formulate relevant treatment plans [5, 6, 18, 20, 30]. We performed a meta-analysis of PCS scores in the two studies [5, 8] using a fixed-effect model, which showed no statistically significant difference in PCS scores after treatment compared with placebo [SMD = 0.11, 95%CI (− 0.12, 0.35), *P* = 0.334 > 0.05]. The two evaluations evaluated and analyzed the measured data of the patients from the physiological and psychological dimensions respectively, which showed no statistical significance, possibly because the symptoms of PTLD patients were mainly non-specific subjective symptoms with great individual differences [11, 21, 24, 30].

BDI is the most widely used tool to measure depression levels. It can be used not only to screen depression, but also to evaluate the severity of depression in patients. It is simple to operate, has good reliability and validity, and can be used for clinical diagnosis [25, 26]. For PTLD patients, their own symptoms and long-term pain will cause psychological damage to them. Therefore, BDI measurement can be used to determine whether the patients' pain is alleviated after antibiotic treatment, thus alleviating their original depression degree [8, 16]. We performed a meta-analysis of BDI scores in the two studies [8, 16] using a random-effects model, which showed no statistically significant difference in BDI scores after treatment compared with placebo [SMD = 0.18, 95%CI (− 0.48, 0.83), *P* = 0.598 > 0.05]. Although studies have shown that the degree of depression of patients will increase with the aggravation of PTLD symptoms, the degree of depression of patients can hardly be alleviated with the alleviation or disappearance of symptoms due to long-term suffering [9], and the common symptoms of PTLD will lead to sleep disorders [28], which will undoubtedly bring different degrees of depression risk to patients.

FSS-11 is the most widely used scale to measure the fatigue severity of the subjects [12]. Fatigue and fatigue are common symptoms of PTLD patients [24], and the long-term persistence of other non-specific subjective symptoms, such as myalgia, arthralgia, sleep disturbance and cognitive and memory impairment [11, 27, 28, 31], will aggravate the feeling of fatigue. Therefore, FSS-11 can be used to clarify the improvement of fatigue after antibiotic treatment [7, 19, 30, 31]. Network meta-analysis of FSS-11 scores of 4 RCTs [5, 8, 16, 19] showed that the FSS scale scores of patients treated with Ceftriaxone for PTLD were better than those treated with doxycycline and placebo, indicating that fatigue symptoms of patients treated with Ceftriaxone for PTLD were improved. Analysis of SUCRA values suggests that ceftriaxone may be the best intervention in antibiotic therapy for PTLD.

It can be seen from the above results that our paper is helpful for drug selection of antibiotic therapy for PTLD, but our limitations include: (1) the number of RCTs is small; (2) The duration and dose of treatment in these RCTs are not uniform; (3) The follow-up time of various RCTs is different to some extent; (4) Fewer RCTs discussed adverse reactions. To sum up, the conclusions we draw may be biased. Therefore, more research on high-quality RCTs for PTLD is needed to help more patients choose therapeutic drugs.

## Conclusion

In a systematic review and meta-analysis, ceftriaxone treatment appeared to be the best option for the treatment of PTLD despite the lack of high-quality evidence, yet the effectiveness of these interventions may vary from individual to individual, so more direct comparative trials are needed to provide more confidence in clinical decision making.

## Supplementary Information

**Additional file 1**. **Table S1**. Basic information of studies included in the meta-analysis. **Table S2**. This figure of AE in post Lyme disease syndrome patients. **Table S3**. The search strategies and specific date of this study.

## Data Availability

The datasets used and analyzed during the current study are available from the corresponding author on reasonable request.

## Abbreviations

PTLD: Post Lyme disease syndrome
BDI: Beck Depression Inventory
MCS: Mental-health scale
PCS: Physical-functioning scale
FSS: Fatigue measure
RCT: Randomized Controlled Trial
