# Supplementary material for: Efficacy and safety of antibiotic therapy for post-Lyme disease? A systematic review and network meta-analysis
Source: BMC Infect Dis. 2023 Jan 12;23:22. doi: 10.1186/s12879-023-07989-4 (PMC9838005; doi:10.1186/s12879-023-07989-4)
Supplement: Supplementary file 1 — Additional file 1. Table S1. Basic information of studies included in the meta-analysis. Table S2. This figure of AE in post Lyme disease syndrome patients. Table S3. The search strategies and specific date of this study. [file 12879_2023_7989_MOESM1_ESM.docx]

| **Study ID (First author)** | **Year of publication** | **Title** | **Methods of diagnosis** | **Therapy duration** | **Time of follow-up** | **Gender（T/C)(M/F)** | **Age（T/C)** | **Symptom** | **Total number** | **Previous antibiotic treatment（T/C)** |
| --- | --- | --- | --- | --- | --- | --- | --- | --- | --- | --- |
| Fallon | 2008 | A randomized, placebo-controlled trial of repeated IV antibiotic therapy for Lyme encephalopathy | with physician documented Lyme borreliosis, with serologic confirmation,current positive IgG western blot, and subjective and objective memory impairment (Wechsler Memory Scale-III) | 70 days | 12or24 weeks | Male & Female（15/22）/(18/13） | Adult (＞18 y)（45.1±13.2）/（45.6±11.3） | patients had mild to moderate cognitive impairment and marked levels of fatigue, pain, and impaired physical functioning | 55 | IV:(2.5±2.0)/(1.9±1.3),oral:(7.9±10.2)/(5.9±7.6) |
| Anneleen Berende | 2016 | Randomized Trial of Longer-Term Therapy for Symptoms  Attributed to Lyme Disease | with symptoms lasting more than 6 months can be attributed to Lyme mycobacteria disease. (Confirmed Lyme disease manifestations or current IgG Western blot positive) | 56 days | 12 weeks | Male & Female（46/40)/(54/42)/(51/47) | Adult (<40 or ≥40 years)(（48.1±12.8）/（48.2±13.0）/（50.0±7.9） | Arthralgia,Musculoskeletal pain,Sensory disturbances,Neuralgia,Neurocognitive symptoms,Fatigu | 280 | IV:(2.0±1.26)/(2.0±1.26)/(2.0±1.11) |
| R.F.Kaplan | 2003 | Cognitive function in post-treatment Lyme disease Do additional antibiotics help? | patients at least 18，with a positive western blot for lgG antibodies against B. burgdorferi antigens and have documentation of an erythema migrans skin lesion provide by an physician. The chronic symptoms had to have begun within 6 months after the initial infection with B.burgdorferi and had to have persisted for at least 6months after treatment of the initial infection but<12 years. | 60 days | 90-day and 180-day | Male & Female(44/43)/(20/28) | Adult (＞18 y)(54.0±13.7)/(51.1±12.0) | All patients had one or more of the following symptoms that interfered with their functioning:wide spread musculoskeletal pain, cognitive impairment, radicular pain, paresthesias, ordysesthesias | 125 | NONE |
| L.B. Krupp | 2003 | Study and treatment of post Lymedisease (STOP-LD) A randomized double masked clinical trial | 1) Erythema migrans or late manifestation of Lyme disease; 2)Completion of standard antibiotic therapy for Lyme disease; 3)Severe fatigue；4）Fatigue was assessed at two independent screenings preceding study enrollment and had to have an onset coincident with the diagnosis of Lyme disease. 5）Confounded the assessment of severe fatigue or cognitive loss were excluded. 6）cephalosporin allergy and severe psychiatric disorders were excluded. 7） Patients identified with a lifetime history of depressive disorders were not excluded. | 28 days | 6 month | Male & Female(13/15)/(13/14) | Adult (18~70 y)48.0 ±11.8 /47.0±9.7 | severe fatigue(defined by FSS-11) | 55 | 6.3±3.2/8.2±9.14 |

**eTable 1**. Basic information of studies included in the meta-analysis.

| **Study ID** | **Adverse events** | | | | | **Adverse events** | | | | | **Adverse events** | | | | |
| --- | --- | --- | --- | --- | --- | --- | --- | --- | --- | --- | --- | --- | --- | --- | --- |
| Anneleen Berende 2016 | **Drug** | **Syptoms** | **Responders** | **Sample size** | **AE** | **Drug** | **Syptoms** | **Responders** | **Sample size** | **AE** | **Drug** | **Syptoms** | **Responders** | **Sample size** | **AE** |
|  | Doxycycline | Diarrhea | 4 | 86 | 4.65% | Clarithromycin– Hydroxychloroquine | Diarrhea | 9 | 96 | 9.38% | Placebo | Diarrhea | 6 | 98 | 6.12% |
|  | Doxycycline | Nausea | 9 | 86 | 10.47% | Clarithromycin– Hydroxychloroquine | Nausea | 10 | 96 | 10.42% | Placebo | Nausea | 5 | 98 | 5.10% |
|  | Doxycycline | Rash | 1 | 86 | 1.16% | Clarithromycin– Hydroxychloroquine | Rash | 8 | 96 | 8.33% | Placebo | Rash | 1 | 98 | 1.02% |
|  | Doxycycline | Mucosal fungal infection | 5 | 86 | 5.81% | Clarithromycin– Hydroxychloroquine | Mucosal fungal infection | 4 | 96 | 4.17% | Placebo | Mucosal fungal infection | 3 | 98 | 3.06% |
|  | Doxycycline | Photosensitivity | 16 | 86 | 18.60% | Clarithromycin– Hydroxychloroquine | Headache | 2 | 96 | 2.08% | Placebo | Photosensitivity | 1 | 98 | 1.02% |
|  | Doxycycline | Dizziness | 3 | 86 | 3.49% | Clarithromycin– Hydroxychloroquine | Dizziness | 5 | 96 | 5.21% | Placebo | Dizziness | 5 | 98 | 5.10% |
|  | Doxycycline | Visual impairment | 1 | 86 | 1.16% | Clarithromycin– Hydroxychloroquine | Visual impairment | 4 | 96 | 4.17% | Placebo | Visual impairment | 4 | 98 | 4.08% |
|  | Doxycycline | Adverse events | 39 | 86 | 45.35% | Clarithromycin– Hydroxychloroquine | Adverse events | 42 | 96 | 43.75% | Placebo | Adverse events | 27 | 98 | 27.55% |
|  |  |  |  |  |  |  |  |  |  |  | Placebo | Headache | 2 | 98 | 2.04% |
| L.B. Krupp 2003 | Ceftriaxone | Anaphylaxis | 1 | 28 | 3.57% | Placebo | IV sepsis | 3 | 27 | 11.11% |  |  |  |  |  |

**eTable 2.** This figure of AE in post Lyme disease syndrome patients.

| **PubMed** | | | |
| --- | --- | --- | --- |
| **Search number** | **Query** | **Results** | **Time** |
| **1** | **"Anti-Bacterial Agents"[Mesh]** | **432879** | **2022-12-16** |
| **2** | **(((antibiotics[Title/Abstract]) OR (antibiotic[Title/Abstract])) OR (antibacterial[Title/Abstract])) OR (antibacterial agents[Title/Abstract])** | **484583** | **2022-12-16** |
| **3** | **("Anti-Bacterial Agents"[Mesh]) OR ((((antibiotics[Title/Abstract]) OR (antibiotic[Title/Abstract])) OR (antibacterial[Title/Abstract])) OR (antibacterial agents[Title/Abstract]))** | **694988** | **2022-12-16** |
| **4** | **"Post-Lyme Disease Syndrome"[Mesh]** | **47** | **2022-12-16** |
| **5** | **((Post-Lyme syndrome) OR (Post-Lyme Disease Syndromes)) OR (Post-Treatment Lyme Disease)** | **337** | **2022-12-16** |
| **6** | **("Post-Lyme Disease Syndrome"[Mesh]) OR (((Post-Lyme syndrome) OR (Post-Lyme Disease Syndromes)) OR (Post-Treatment Lyme Disease))** | **337** | **2022-12-16** |
| **7** | **(("Anti-Bacterial Agents"[Mesh]) OR ((((antibiotics[Title/Abstract]) OR (antibiotic[Title/Abstract])) OR (antibacterial[Title/Abstract])) OR (antibacterial agents[Title/Abstract]))) AND (("Post-Lyme Disease Syndrome"[Mesh]) OR (((Post-Lyme syndrome) OR (Post-Lyme Disease Syndromes)) OR (Post-Treatment Lyme Disease)))** | **185** | **2022-12-16** |
| **Web of Science** | | | |
| **No.** | **Query** | **Results** | **Time** |
| **#1** | **TS=(Anti-Bacterial Agents) OR TS=(antibiotics) OR TS=(antibiotic) OR TS=(antibacterial) OR TS=(antibacterial agents)** | **1534443** | **2022-12-16** |
| **#2** | **TS=(Post-Lyme Disease Syndrome) OR TS=(Post-Lyme Disease Syndromes) OR TS=(Post-Treatment Lyme Disease) OR TS=(Post-Lyme syndrome)** | **358** | **2022-12-16** |
| **#1 AND #2** |  | **206** | **2022-12-16** |
| **Embase** | | | |
| **No.** | **Query** | **Results** | **Time** |
| **#1** | **'antiinfective agent'/exp** | **4,564,991** | **2022-12-16** |
| **#2** | **antibiotics:ab,ti OR antibiotic:ab,ti OR antibacterial:ab,ti OR 'antibacterial agents':ab,ti OR 'anti-bacterial agents':ab,ti** | **632,148** | **2022-12-16** |
| **#3** | **#1 OR #2** | **4,696,398** | **2022-12-16** |
| **#4** | **'post-lyme disease syndrome'/exp** | **71** | **2022-12-16** |
| **#5** | **'post-lyme syndrome':ab,ti OR 'post-lyme disease syndromes':ab,ti OR 'post-treatment lyme disease':ab,ti** | **130** | **2022-12-16** |
| **#6** | **#4 OR #5** | **162** | **2022-12-16** |
| **#7** | **#3 AND #6** | **108** | **2022-12-16** |
| **Cochrane Library** | | | |
| **ID** | **Search** | **Hits** | **Time** |
| **#1** | **MeSH descriptor: [Anti-Bacterial Agents] explode all trees** | **13163** | **2022-12-16** |
| **#2** | **(antibiotics):ti,ab,kw OR (antibiotic):ti,ab,kw OR (antibacterial):ti,ab,kw OR (antibacterial agents):ti,ab,kw (Word variations have been searched)** | **41994** | **2022-12-16** |
| **#3** | **#1 OR #2** | **43007** | **2022-12-16** |
| **#4** | **MeSH descriptor: [Post-Lyme Disease Syndrome] explode all trees** | **5** | **2022-12-16** |
| **#5** | **(Post-Lyme syndrome):ti,ab,kw OR (Post-Lyme Disease Syndromes):ti,ab,kw OR (Post-Treatment Lyme Disease):ti,ab,kw** | **28** | **2022-12-16** |
| **#6** | **#4 OR #5** | **28** | **2022-12-16** |
| **#7** | **#3 AND #6** | **22** | **2022-12-16** |

**eTable 3.** The search strategies and specific date of this study.
